# Supplementary figures and images for: Spinopelvic Motion Evaluation in Patients Undergoing Total Hip Arthroplasty and Patient-Specific Target for Acetabular Cup Placement
Source: J Pers Med. 2024 Dec 19;14(12):1161. doi: 10.3390/jpm14121161 (PMC11678274; doi:10.3390/jpm14121161)

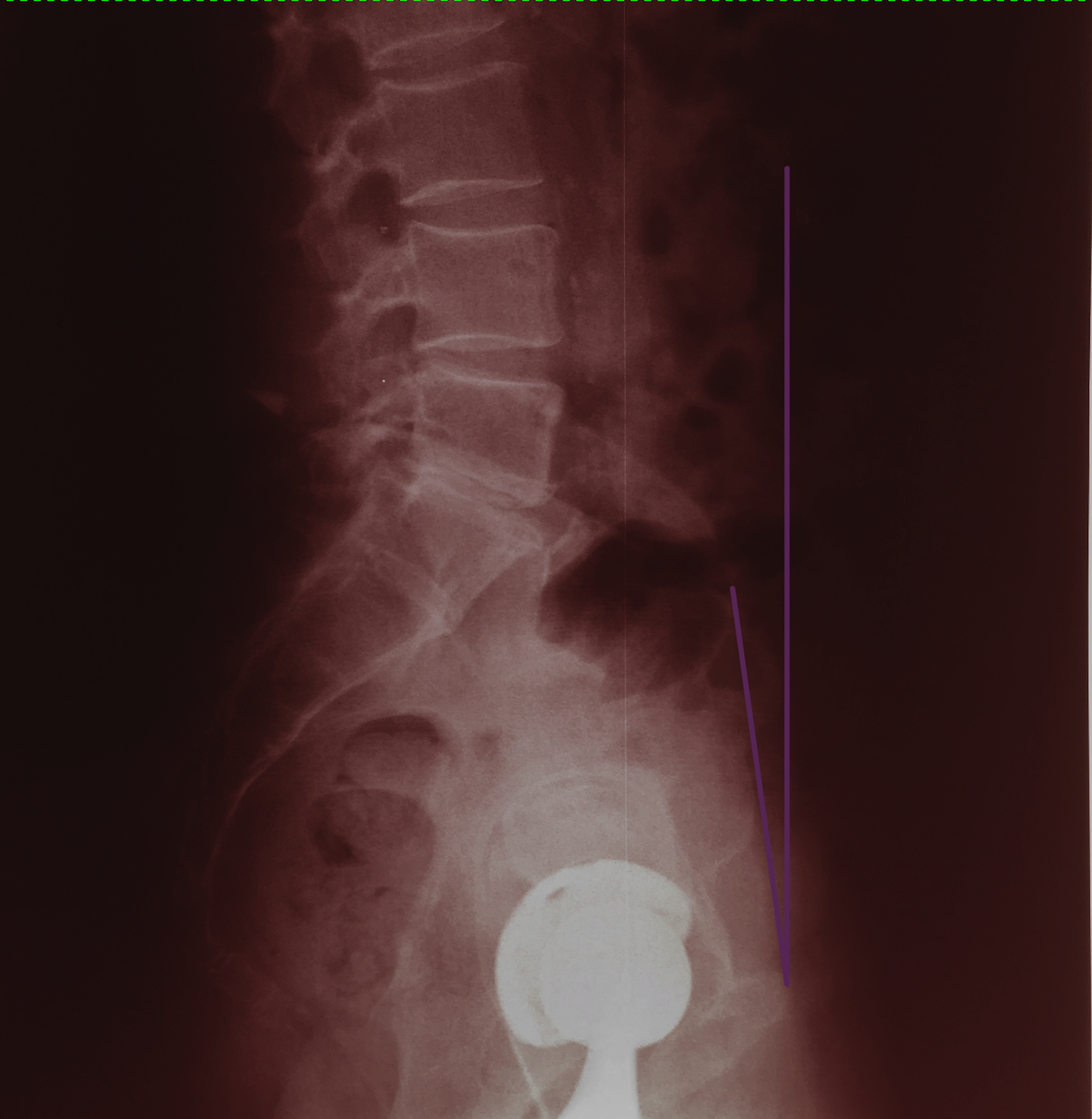

Supplement: Supplementary file 1 [file jpm-14-01161-s001.zip › Figure S1.png]

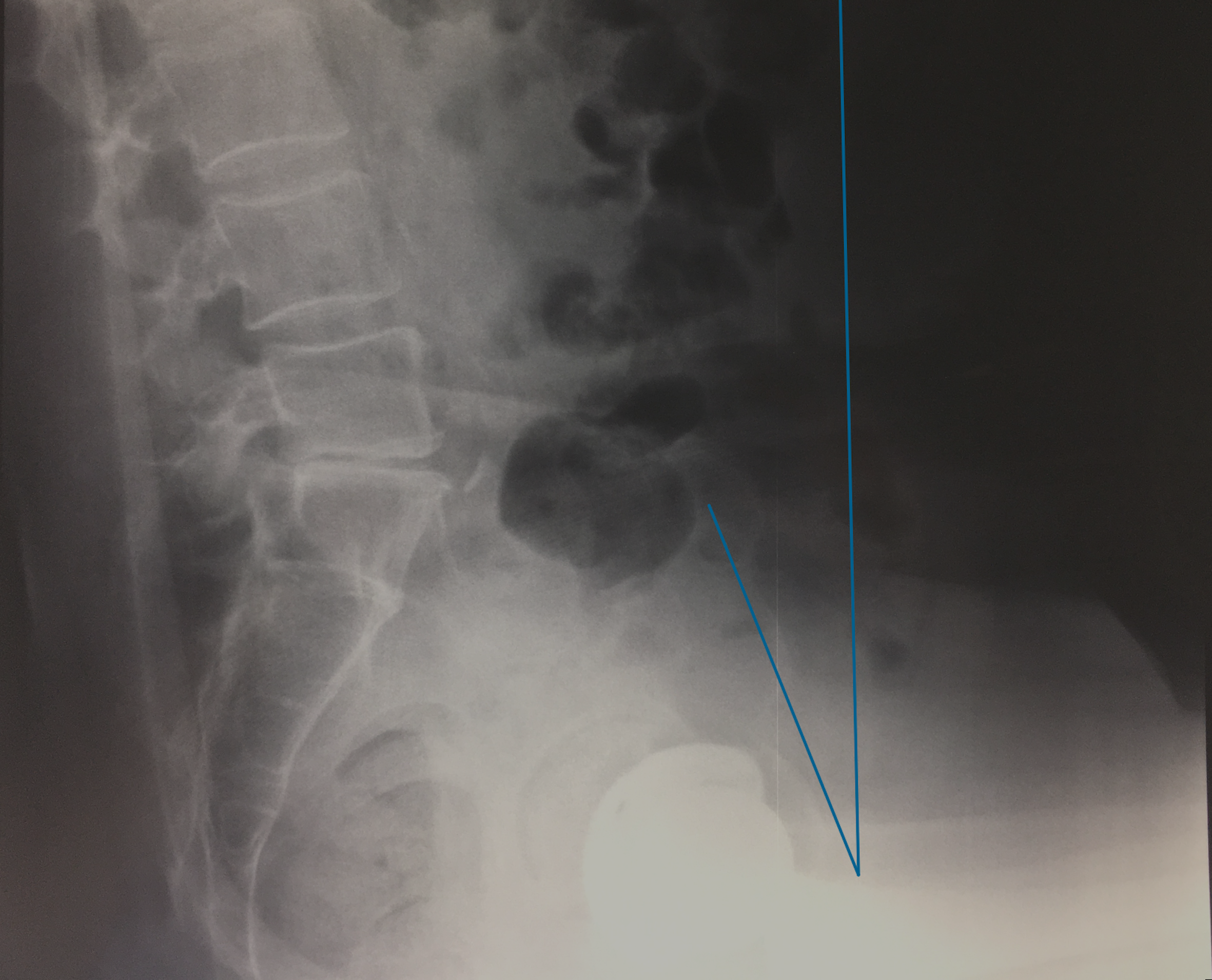

Supplement: Supplementary file 1 [file jpm-14-01161-s001.zip › Figure S2.png]

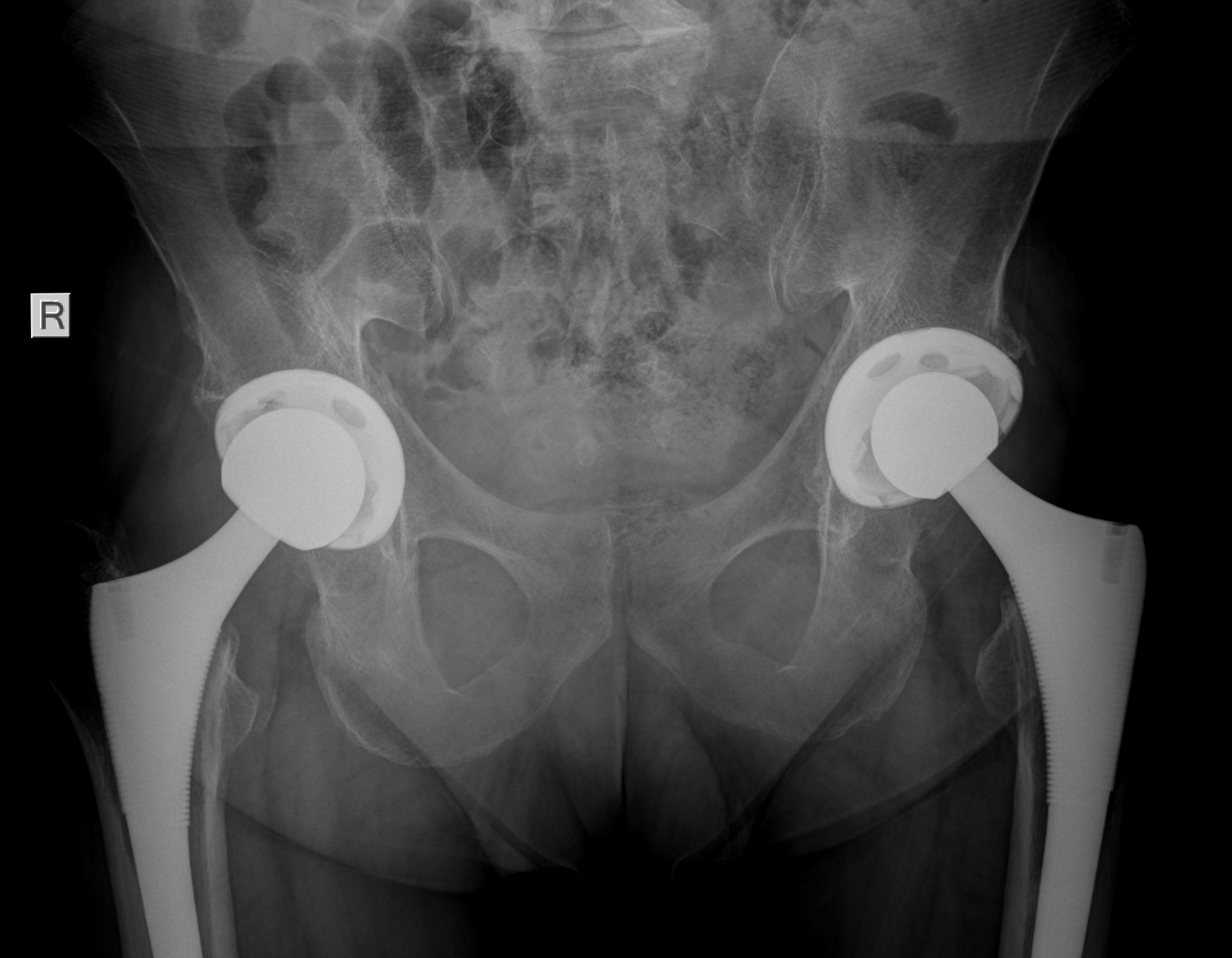

Supplement: Supplementary file 1 [file jpm-14-01161-s001.zip › Figure S3.png]
